# Organic optoelectronics creating new opportunities for science and applications

**DOI:** 10.1007/s12200-022-00052-1

**Published:** 2022-12-26

**Authors:** Yinhua Zhou, Chengliang Wang

**Affiliations:** 1 Wuhan National Laboratory for Optoelectronics, Huazhong University of Science and Technology, Wuhan, 430074 China; 2 School of Integrated Circuits, Huazhong University of Science and Technology, Wuhan, 430074 China; 3 School of Optical and Electronic Information, Huazhong University of Science and Technology, Wuhan, 430074 China

In the year of 2000, the Nobel prize in chemistry was awarded to three professors, Alan Heeger, Alan MacDiarmid, and Hideki Shirakawa, for their contributions to discovery and development of conducting polymers. Their works altered the previously-held idea that organic and polymer materials are electrically insulating. Instead, conjugated organic or polymeric materials can be conductors and semiconductors. Compared with traditional inorganic counterparts, organic conductors and semiconductors have advantages of excellent mechanical flexibility, easy processing, potentially low cost, recyclability and easy tuning of their optoelectronic properties by molecular tailoring. In the past years, various organic optoelectronic devices have been demonstrated, including organic light-emitting diodes (OLED), organic photovoltaics (OPV), organic photodiodes (OPD), organic field-effect transistors (OFET), organic energy storage devices, etc. OLEDs have become mature technology for displays and lighting. Other organic optoelectronic devices are still being researched for practical applications. Novel organic optoelectronic materials, physics, devices and applications are ripe for investigation and exploration for a more sustainable society.

This special issue on “Organic Optoelectronics” contains eight papers. Three of them are review articles, the other five are four research article and one letter. Li et al. [1] reviewed fabrications, structures and properties of β-ketoenamine-based covalent organic frameworks (COF). Their applications in fluorescence sensors, energy storage, photocatalysis, electrocatalysis, and batteries were also reviewed. Sun et al. [2] reviewed recent advances of high-performance organic hole-transporting materials for inverted perovskite solar cells. Shan et al. [3] reviewed the fundamentals of the device structure and operation mechanisms of OPD. Recent advances of performance enhancement strategies and applications of OPDs were also reviewed. Kim et al. [4] reported OPD devices with dual functions of indoor photovoltaic conversion and high-speed photodetection. Chen et al. [5] reported that band-like transport in a nonfullerene acceptor semiconductor of Y6, which is a key material in OPV application. The results here are important for understanding the high-power conversion efficiency produced by active layers with Y6. Jiang et al. [6] reported 7,7′,8,8′-tetracyanoquinodimethane (TCNQ)-based organic cocrystals with red emission and n-type charge transport. Liu et al. [7] adopted two typical organic semiconductors as interfacial modification layers in sodium metal batteries, providing guidance for designing functional interfaces in batteries. Xue et al. [8] reported a thermally activated delayed fluorescence (TADF) small molecule bis-[3-(9,9-dimethyl-9,10-dihydroacridine)-phenyl]-sulfone (*m*-ACSO2) that could be used as a universal host material for sensitizing conventional fluorescent polymers to substantially enhance their external quantum efficiency.

The eight papers contained in the issue cover materials, their underlying physics, and their device applications. The devices include OPD, OFET, OPV, perovskite PV, and batteries. We believe these contents are comprehensive, interdisciplinary and inspiring, and can stimulate more cutting-edge work in the community of organic functional materials and organic optoelectronics.
